# Metabolic Plasticity and Combinatorial Radiosensitisation Strategies in Human Papillomavirus-Positive Squamous Cell Carcinoma of the Head and Neck Cell Lines

**DOI:** 10.3390/cancers13194836

**Published:** 2021-09-28

**Authors:** Mark D. Wilkie, Emad A. Anaam, Andrew S. Lau, Carlos P. Rubbi, Nikolina Vlatkovic, Terence M. Jones, Mark T. Boyd

**Affiliations:** 1 Cancer Research Centre, Department of Molecular & Clinical Cancer Medicine, The University of Liverpool, 200 London Road, Liverpool L3 9TA, UK; E.Anaam@liverpool.ac.uk (E.A.A.); andrew.lau@liverpool.ac.uk (A.S.L.); C.Rubbi@liverpool.ac.uk (C.P.R.); Vlatko@liverpool.ac.uk (N.V.); T.M.Jones@liverpool.ac.uk (T.M.J.); mboyd@liverpool.ac.uk (M.T.B.); 2 Department of Otorhinolaryngology–Head & Neck Surgery, University Hospital Aintree, Lower Lane, Liverpool L9 7AL, UK

**Keywords:** p53, cancer, glycolysis, oxidative phosphorylation, metabolism, head and neck cancer, human papillomavirus

## Abstract

**Simple Summary:**

A subset of head and neck cancers (SCCHN) are caused by human papillomavirus (HPV). As these tumours tend to affect younger patients and are associated with favourable survival, there is a pressing need to find ways to reduce long-term treatment toxicity while maintaining oncological efficacy. We studied utilisation of metabolic pathways in HPV-positive SCCHN cells with the aim of exploiting such for potential therapeutic benefit. We found that these tumours maintained metabolic diversity, in contrast to what we have observed in traditional SCCHN cells associated with mutations in the *TP53* gene. This, in turn, correlated with susceptibility to metabolic inhibitors, insofar as a combination of these agents acting on different metabolic pathways was required to augment the effects of ionising radiation (a mainstay of treatment for SCCHN). Notionally, this may provide a means of treatment de-intensification by facilitating radiation dose reduction to minimise the impact of treatment on long-term function.

**Abstract:**

Background: A major objective in the management of human papillomavirus (HPV)-positive squamous cell carcinoma of the head and neck (SCCHN) is to reduce long-term functional ramifications while maintaining oncological outcomes. This study examined the metabolic profile of HPV-positive SCCHN and the potential role of anti-metabolic therapeutics to achieve radiosensitisation as a potential means to de-escalate radiation therapy. Methods: Three established HPV-positive SCCHN cell lines were studied (UM-SCC-104, UPCI:SCC154, and VU-SCC-147), together with a typical *TP53* mutant HPV-negative SCCHN cell line (UM-SCC-81B) for comparison. Metabolic profiling was performed using extracellular flux analysis during specifically designed mitochondrial and glycolytic stress tests. Sensitivity to ionising radiation (IR) was evaluated using clonogenic assays following no treatment, or treatment with: 25 mM 2-deoxy-D-glucose (glycolytic inhibitor) alone; 20 mM metformin (electron transport chain inhibitor) alone; or 25 mM 2-deoxy-D-glucose and 20 mM metformin combined. Expression levels of p53 and reporters of p53 function (MDM2, p53, Phospho-p53 [Ser15], TIGAR and p21 [CDKN1A]) were examined by western blotting. Results: HPV-positive SCCHN cell lines exhibited a diverse metabolic phenotype, displaying robust mitochondrial and glycolytic reserve capacities. This metabolic profile, in turn, correlated with IR response following administration of anti-metabolic agents, in that both 2-deoxy-D-glucose and metformin were required to significantly potentiate the effects of IR in these cell lines. Conclusions: In contrast to our recently published data on HPV-negative SCCHN cells, which display relative glycolytic dependence, HPV-positive SCCHN cells can only be sensitised to IR using a complex anti-metabolic approach targeting both mitochondrial respiration and glycolysis, reflecting their metabolically diverse phenotype. Notionally, this may provide an attractive platform for treatment de-intensification in the clinical setting by facilitating IR dose reduction to minimise the impact of treatment on long-term function.

## 1. Introduction

Squamous cell carcinoma of the head and neck (SCCHN) is the sixth most common cancer globally, with an estimated incidence of 750,000 cases per year [1]. Whilst tobacco and alcohol consumption are traditional risk factors [2], there is also now a considerable body of evidence implicating human papillomavirus (HPV) as a cause of SCCHN, in particular of oropharyngeal SCC affecting the base of tongue and tonsils [3].

Although rates of oral and laryngeal cancers are stable or decreasing slightly in developed countries, primarily because the population is smoking less, there has been a dramatic upsurge in the incidence of oropharyngeal cancer in the developed world in recent years. In the United States (US), the incidence of oropharyngeal SCC increased by 22% between 1999 and 2006, after no change between 1975 and 1999 [4], and the United Kingdom (UK) has seen a doubling in incidence from 1/100,000 population to 2.3/100,000 in just over a decade [5]. This rapid increase has been widely attributed to an exponential rise in the incidence of HPV-related disease, a consensus corroborated by several prevalence studies [6,7].

HPV-positive SCCHN is associated with favourable survival outcomes irrespective of the treatment modality employed [8,9]. This, together with the fact that HPV-driven disease tends to affect younger and generally medically fitter patients, who are therefore likely to experience the functional ramifications of their treatment long-term, has led many to propose treatment de-intensification [10]. Consequently, a key objective in translational research is to identify ways of sensitising these tumours to the effects of current treatments, not only to improve efficacy, but also to minimise the substantial toxic effects. Fundamental to this is to elucidate the cellular processes that may determine radio- and/or chemo-sensitivity to facilitate therapeutic targeting of the key pathways that may impinge on these processes. 

Alteration of cellular metabolism is now widely considered to be a hallmark of the cancer phenotype, intrinsic to malignant transformation [11], and as such presents a potentially attractive therapeutic target. However, whilst disruption of metabolic circuitry may occur to some degree in all tumours, there is undoubtedly heterogeneity, which may reflect both tissue-specific effects and distinct oncogenic events driving tumorigenesis in different tumour types [12]. Detailed study and consideration of the metabolic phenotype of individual cancers is paramount, therefore, if effective therapeutic strategies targeting metabolism are to be developed and effectively deployed. 

In the context of HPV-negative SCCHN, we and others have shown previously that metabolic phenotype and associated therapeutic opportunities are dictated by *TP53* status, with mutational loss of wild-type p53 function conferring a metabolic switch away from oxidative phosphorylation and towards glycolytic dependence [13,14,15]. Whilst HPV-positive tumours rarely harbour *TP53* mutations (2–3% of cases [16]), the importance of the HPV E6 oncoprotein in targeting p53 for proteasomal degradation in HPV oncogenesis [17,18], invites scrutiny of metabolism in HPV-positive disease, particularly given the dearth of previously published data. The aims of this study, therefore, were to examine the metabolic profile in HPV-positive SCCHN and to determine whether anti-metabolic therapy might be employed to potentiate the effects of ionising radiation (IR).

## 2. Methods

### 2.1. Cell Lines and Culture Conditions 

The following established immortalised cell lines were used in this study: UM-SCC-1, UM-SCC-81B, and UM-SCC-104 (kindly provided by Professor T.E. Carey, University of Michigan, Ann Arbor, MI, USA); UPCI:SCC154 (kindly provided by Professor S. Gollin, University of Pittsburgh, Pittsburgh, PA, USA); and VU-SCC-147 (kindly provided by Dr Josephine Dorsman, VU University Medical Centre, Amsterdam, The Netherlands). UM-SCC-104, UPCI:SCC154, and VU-SCC-147 are all SCCHN cell lines reported to be HPV-positive [19,20]. UM-SCC-1, a SCCHN cell line known to be p53 null [21], was used as a negative control in p53 Western blot analysis, while UM-SCC-81B, an HPV-negative oropharyngeal SCC cell line known to harbour a *TP53* mutation (H193R) [22], was used for comparative purposes in metabolic profiling and clonogenic survival experiments being typical of the lines we recently described [15]. All cell lines were confirmed as identical to their published genotype [23,24,25] via short tandem repeat (STR) analysis with GenePrint^®^ 10 System (Promega, Southampton, UK) and screened and confirmed negative for mycoplasma contamination using the e-Myco^TM^ mycoplasma PCR detection kit (ChemBio, Luton, UK). Cells lines were grown in Nunc^TM^ cell culture treated flasks with filter caps (Thermo Fisher Scientific, Paisley, UK) in a humidified cell incubator at 37 °C with 5% CO_2_ and, with the exception of UPCI:SCC154, were maintained in Dulbecco’s modified Eagle’s media (DMEM, Sigma-Aldrich, Gillingham, UK) supplemented with 10% fetal bovine serum (FBS), 1% penicillin/streptomycin, 1% L-glutamine, and 1% non-essential amino acids. UPCI:SCC154 was maintained in Eagle’s minimum essential medium supplemented with 15% FBS, with additional supplements as above.

### 2.2. Western Blotting

Cells were harvested either untreated or 8 h after irradiation at a dose of 6Gy. Proteins extracted as described previously [26]. Typically, 50 μg samples of total protein were separated by SDS–PAGE and transferred to Immun-Blot^®^ PVDF Membrane (Bio-Rad, Hercules, CA, USA). Western blotting was performed as described previously [23]. Mouse monoclonal antibodies against MDM2 (IF-2) (Calbiochem, San Diego, CA, USA), p53 (DO-1) (Calbiochem), TIGAR (E-2) (Santa Cruz, Dallas, TX, USA), p21 (*CDKN1A)* (F-5) (Santa Cruz), and a rabbit polyclonal antibody against Phospho-p53 (Ser15) (#9284) (Cell Signaling Technology, Danvers, MA, USA) were used to probe the expression of those proteins. Detection of Vinculin, using a mouse monoclonal antibody (V9131) (Sigma-Aldrich), was included as a loading control. Secondary antibodies were sheep anti-mouse (RPN4201) and donkey anti-rabbit (NA934) (GE Healthcare, Little Chalfont, UK) used at dilutions of 1:2500 and 1:5000 respectively. Membranes were washed with PBS-0.1% (*v*/*v*) Tween^®^-20 for all proteins except for phospho-p53 (Ser15) where the membrane was washed with TBS-0.1% (*v*/*v*) Tween^®^-20. Bio-Rad EveryBlot Blocking Buffer was used to suppress non-specific antibody binding. Signals were detected by chemiluminescence using Clarity Western ECL Substrate (Bio-Rad) and recorded using ChemiDoc MP imaging system (Bio-Rad).

### 2.3. Metabolic Profiling Studies 

Metabolic studies were performed using an XF24 analyser (Seahorse Bioscience, Copenhagen, Denmark) to undertake either mitochondrial or glycolytic stress tests, essentially according to the manufacturer’s instructions. Experimental readouts are oxygen consumption rate (OCR) in pmol/min and extracellular acidification rate (ECAR) in mpH/min, to provide surrogate measures of oxidative phosphorylation and glycolysis respectively. Data were analysed using Wave software (Seahorse Bioscience). During mitochondrial stress tests, inhibitors were injected sequentially as follows: oligomycin 1.25 μM, FCCP 1.5 μM, and lastly a combination of rotenone and antimycin-A 1 μM. During glycolytic stress tests, inhibitors were injected sequentially as follows: glucose 10 mM, oligomycin 1.25 μM, and finally 2-DG 50 mM. ECAR was measured in mpH/min. 

To enable quantification and for comparison of OCR and ECAR metabolic measurements between cell lines, normalisation to sample DNA content was employed. The DNA content of samples following completion of mitochondrial and glycolytic stress tests was determined in Corning^®^ 96-well black bottom plates (Sigma-Aldrich) and then DNA content was measured using a CyQUANT^®^ cell proliferation assay kit (Invitrogen, Paisley, UK), according to the manufacturer’s instructions. Fluorescence was measured at 520 nm following excitation at 508 nm using a POLARstar Omega plate reader (BMG LABTECH, Ortenberg, Germany).

When analysing data between different cell lines non-parametric statistics were used due to the number of samples in each group (<30) as per central limit theorem. Specifically, Mann-Whitney *U* tests were utilised and the significance level for all tests was set at 0.05. For the purposes of meaningful and valid comparison analysis of metabolic parameters derived from mitochondrial and glycolytic stress tests was performed for the HPV-positive cell lines grouped together and compared with the *TP53* mutant HPV-negative cell line (UM-SCC-81B). 

### 2.4. Clonogenic Survival Assays 

Clonogenic survival assays were performed using a “plating after treatment” method as described previously [27]. Data were analysed as follows: the plating efficiency (PE) was calculated from the 0 Gy condition by dividing the number of colonies formed by the number of cells seeded. The number of colonies formed after treatment was then used to calculate the surviving fraction (SF) for each treatment condition, accounting for the plating efficiency: SF = (number of colonies formed/number of cells seeded) × PE. Survival parameters to generate treatment-dose survival curves for treatment conditions were then derived from fitting the data by weighted, stratified, linear regression according to the linear–quadratic formula S(D) = exp(αD + βD^2^), where S is survival following a given dose (D) of IR, which also allowed comparison between treatment conditions as described by Franken et al. [27] Specifically, the model summary examined whether the data scatter across two treatment-dose survival curves was described best with one modelled survival curve (null hypothesis) or was best described by two distinct modelled curves (test hypothesis) (i.e., a statistically significant difference [*p* < 0.05] between the compared treatment-dose survival curves) [27].

## 3. Results

### 3.1. HPV-Positive SCCHN Cells Maintain Metabolic Diversity 

**Figure 1:**
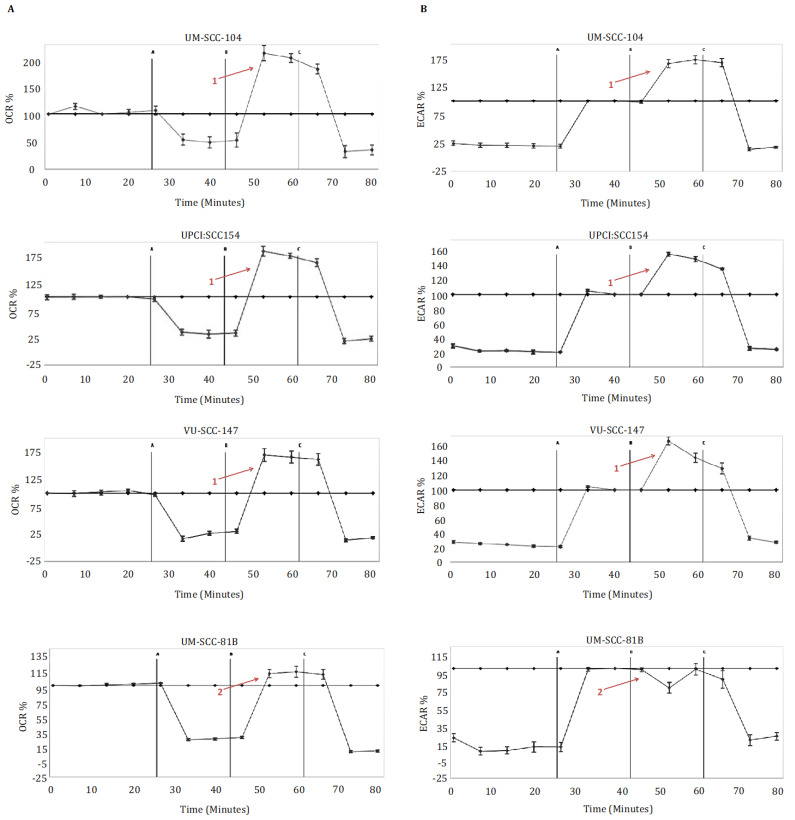
Metabolic profiling in HPV-positive SCCHN cell lines and for comparison, a typical *TP53* mutant HPV-negative line (UM-SCC-81B). Cells were subjected to mitochondrial and glycolytic stress tests (panels (**A**,**B**) respectively). During mitochondrial stress tests, oligomycin 1.25 μM (point A), FCCP 1.5 μM (point B), and rotenone and antimycin-A 1 μM (point C) were sequentially injected, and OCR (pmol/min) measured. During glycolytic stress tests, glucose 10 mM (point A), oligomycin 1.25 μM (point B), and 2-DG 50 mM (point C) were sequentially injected, and ECAR (mpH/min) measured. Data is presented as percentage increases or decreases in OCR and ECAR relative to baseline measurements. The baseline is shown as the black line on the graphs. HPV-positive SCCHN cell lines maintained marked spare respiratory capacities and glycolytic reserves, in contrast to UM-SCC-81B, which exhibited reduced spare respiratory capacity and reduced glycolytic reserved (contrast arrows labelled 1 and 2). Error bars represent standard error of the mean (SEM).

Metabolic profiling of the HPV-positive SCCHN cell lines (UM-SCC-104, UPCI:SCC 154, VU-SCC-147) demonstrated that these cells exhibit a diverse metabolic phenotype, displaying both robust mitochondrial and robust glycolytic reserve capacities (Figure 1). In contrast, and in accordance with what we and others have shown previously [13,14,15], the HPV-negative SCCHN mutant *TP53* SCCHN cell line (UM-SCC-81B) displayed markedly reduced mitochondrial and glycolytic reserves, functioning near or at capacity under basal conditions (Figure 1). FCCP doses were also titrated to ensure the reduced mitochondrial reserve was not related to suboptimal FCCP dosing (Supplementary Figure S1).

**Figure 2:**
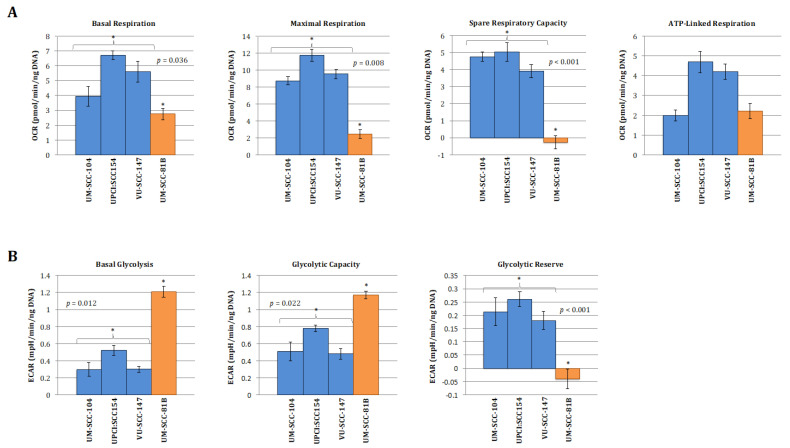

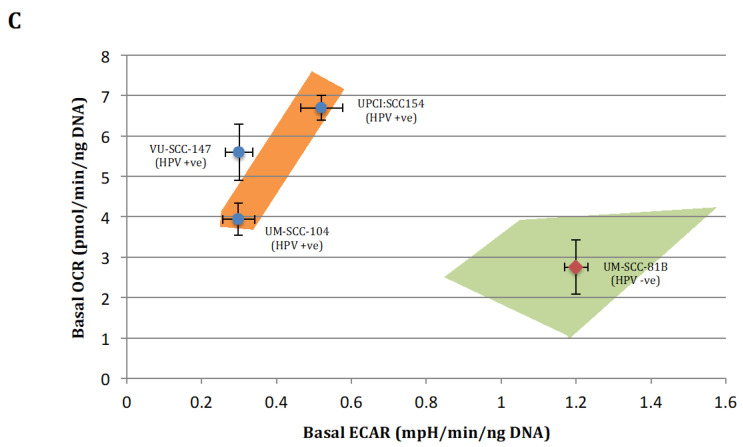
Quantitative OCR and ECAR data, normalised to DNA content for HPV-positive SCCHN cell lines and for comparison, a typical *TP53* mutant HPV-negative line (UM-SCC-81B). As stated in the methods, statistical comparison of absolute values for metabolic parameters was performed for the HPV-positive cell lines grouped together and compared with the *TP53* mutant HPV-negative cell line. (**A**) displays data from mitochondrial stress tests with statistical analysis as follows: basal respiration * *p* = 0.036; maximal respiration * *p* = 0.008; spare respiratory capacity * *p* < 0.001; ATP-linked respiration *p* = non-significant. (**B**) displays data from glycolytic stress tests with statistical analysis as follows: basal glycolysis * *p* = 0.012; glycolytic capacity * *p* = 0.022; glycolytic reserve * *p* < 0.001. (**C**) Plot of relative basal utilisation of mitochondrial respiration and glycolysis. Normalised basal values from glycolytic and mitochondrial stress tests for all cell lines plotted against each other. There is clear grouping of the HPV-positive SCCHN cell lines and the mutant *TP53* cell line, with the former displaying high levels of mitochondrial respiration relative to glycolysis and the latter the opposite. Blue diamonds represent HPV-positive SCCHN cell lines and the red diamond represents the mutant *TP53* cell line. The green and orange shaded areas represent the respective distributions of mutant *TP53* and wild-type SCCHN cell lines as we have demonstrated previously [15]. For all panels error bars represent SEM for each measurement.

To allow for a clearer interpretation of the overall metabolic picture comparison of normalised absolute values for the metabolic parameters generated from the mitchondrial and glyolcytic stress tests was also performed and are depicted in Figure 2. In keeping with the clear patterns shown in the relative stress test plots in Figure 1, the absolute values for maximal respiration, spare respiratory capacity, maximal glycolysis, and glycolytic reserve were significantly greater in the HPV-positive cell lines compared with HPV-negative mutant *TP53* cell line (Figure 2A,B). Interestingly, however, capturing absolute OCR and ECAR values revealed that HPV-positive cells exhibited discernibly higher rates of mitochondrial respiration and lower rates of glycolysis under basal conditions, findings which were borne out more definitively when analysing the relative basal utilisation of mitochondrial respiration and glycolysis (Figure 2C).

Taken together, these findings suggest that HPV-positive SCCHN (and by association p53 wild-type [16]) cells predominantly catabolise glucose through oxidative phosphorylation under basal conditions and maintain robust mitochondrial function, enabling these cells to mount a maximal increase in ETC activity when exposed to mitochondrial stressors. In contrast, in the context of mutational inactivation of wild-type p53 function mitochondrial function appears to be compromised as cells display reduced oxidative phosphorylation under basal conditions and are unable to mount an increase in activity in response to mitochondrial stressors. Glycolysis seemingly predominates with significantly heightened basal levels, which are elevated to maximal cellular capacity leaving little or no glycolytic reserve. This is again consistent with what we and others have shown previously in HPV-negative SCCHN cell lines divergent on TP53 status [13,14,15].

### 3.2. HPV-Positive SCCHN Metabolic Diversity May Be Related to Residual Wild-Type p53 Function

That HPV-positive SCCHN cells maintain metabolic diversity was perhaps surprising given that previous data has shown loss of wild-type p53 function to be associated with a metabolic switch away from mitochondrial respiration and towards glycolytic dependence [13,14,15], and that targeting p53 for proteasomal degradation is key to HPV oncogenesis [17,18]. This prompted us to examine expression of p53 and reporters of p53 function (MDM2, p53, Phospho-p53 [Ser15], TIGAR and p21 [*CDKN1A*]) in the HPV-positive SCCHN cell lines, with the hypothesis that there may be residual wild-type p53 function sufficient to prevent metabolic re-programming.

**Figure 3:**
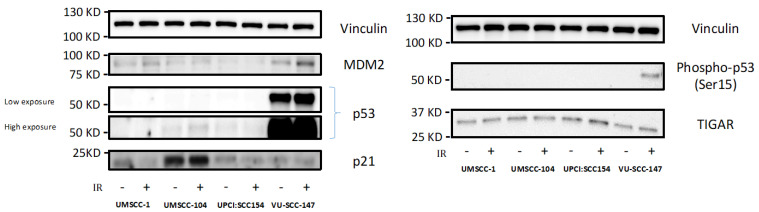
Expression levels of p53 and reporters of p53 function in HPV-positive SCCHN cell lines and for comparison, an endogenously p53-null SCCHN cell line. Western blotting revealed VU-SCC-147 to express p53 under basal conditions, but not so in the cases of UM-SCC-104 and UPCI:SCC 154. As VU-SCC-147 was observed to express high levels of p53 both high and low exposure blots are shown for clarity. Exposure to p53-activating genotoxic stress (6Gy IR [denoted by +]) also induced p53 stabilisation and activation of p53 reporters (specifically Phospho-p53 [Ser15]) in VU-SCC-147. Again, this was not demonstrated convincingly for UM-SCC-104 and UPCI:SCC 154. UM-SCC-1, a SCCHN cell line known to be endogenously p53 null, was included for comparison.

Accordingly, western blotting revealed VU-SCC-147 to express p53 under basal conditions, and more importantly exposure to p53-activating genotoxic stress (6Gy IR) induced p53 stabilisation and activation of p53 reporters in this cell line (Figure 3 [original western bots are shown in Supplementary Figure S2]). This, however, was not demonstrated convincingly for the other HPV-positive SCCHN cell lines (UM-SCC-104 and UPCI:SCC 154). Consistent with this, such expression patterns have also been reported previously for these HPV-positive SCCHN cell lines [28,29].

### 3.3. HPV-Positive SCCHN Cells Require a Combinatorial Anti-Metabolic Therapeutic Approach

Having shown that HPV-positive SCCHN cell lines exhibit a diverse metabolic phenotype in keeping with functional wild-type p53, we sought to evaluate the potential of anti-metabolic approaches that could be used therapeutically. Cells were exposed to IR following no treatment, 2-DG alone, metformin alone, or combined 2-DG and metformin treatment. 2-DG is a stable glucose analogue and a well-recognised glycolytic inhibitor that interferes with glycolysis by inhibiting the glycolytic enzymes phosphoglucose isomerase and hexokinase [30,31]. Metformin has demonstrated activity against mitochondrial respiration, specifically against electron transport chain (ETC) complex I [32], and thus represents an appropriate choice to combine with 2-DG to enable targeting of both glycolysis and mitochondrial respiration.

**Figure 4:**
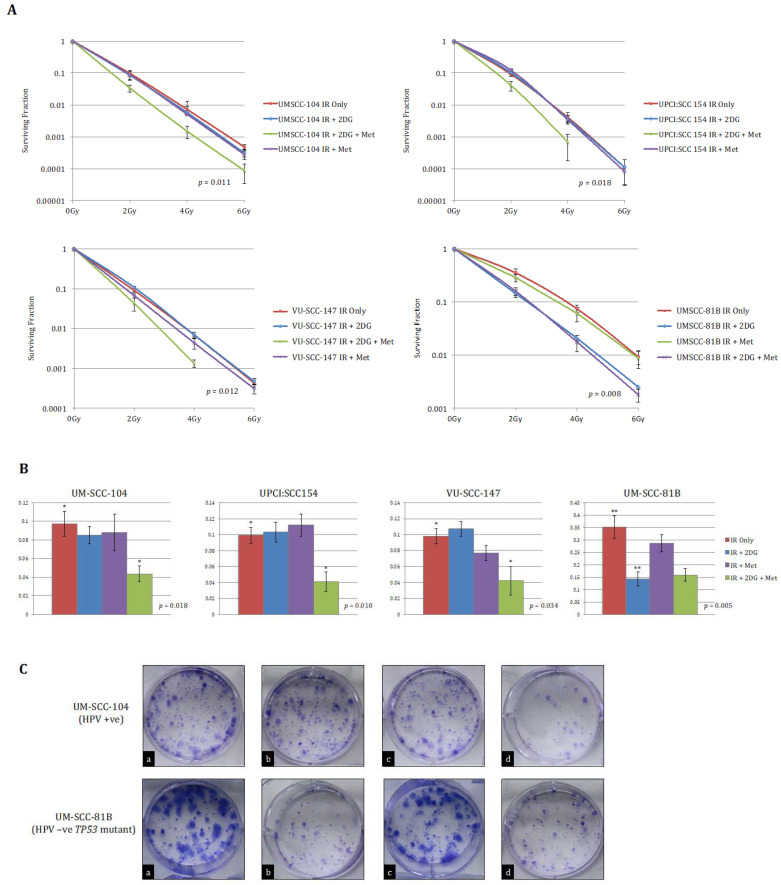
Clonogenic survival data for HPV-positive SCCHN cell lines and for comparison, a typical *TP53* mutant HPV-negative line (UM-SCC-81B). A stated in the methods, clonogenic assays were performed on the indicated SCCHN cell lines exposed to the indicated doses of IR. Cells were either left untreated or pre-treated with 25 mM 2-DG, 20 mM metformin (met), or combined treatment for one hour prior to irradiation. (**A**) displays clonogenic survival curves for each of the SCCHN cell lines and (**B**) the survival fractions at 2 Gy for each indicated treatment condition. Whilst 2-DG or metformin alone failed to have any significant effect on radiation response in any of the HPV-positive cell lines, the combination of 2-DG and metformin resulted in a significant reduction in clonogenic survival (in the case of the HPV-positive cell lines *p*-values represent the difference between the survival curves for the untreated condition and when combined pre-treatment with 2-DG and metformin was administered). In contrast, the addition of 2-DG alone consistently potentiated the effects of IR in UM-SCC-81B cells, while the addition of metformin has no discernible effect on clonogenic survival (the *p*-value in this case represents the difference between the survival curves for the untreated condition and when pre-treatment with 2-DG alone was administered). The results shown represent the mean values obtained from at least three separate experiments and error bars represent the SEMs. * *p* < 0.05, ** *p* < 0.005. (**C**) depicts representative example images of colony formation at 2 Gy for each treatment condition (a = IR alone; b = IR +2-DG; c = IR + 2-DG + Metformin; d = IR + Metformin) for the indicated cell lines.

Accordingly, we predicted that HPV-positive SCCHN cells would display unaltered sensitivity to IR if only glycolysis or the ETC were inhibited, since the cells retain the metabolic flexibility to adapt to these inhibitors, in contrast to our recent findings in *TP53* mutant HPV-negative SCCHN cells, in which metabolic restriction allowed for radiosensitisation by 2-DG alone [15]. Moreover, we hypothesised that the drug combination might radiosensitise these metabolically more flexible cells. As expected, whilst 2-DG or metformin alone failed to have any effect on IR response, the combination of 2-DG and metformin resulted in significant potentiation of IR effects (Figure 4). Again, for comparative purposes anti-metabolic therapeutic approaches were evaluated in UM-SCC-81B as a typical representative *TP53* mutant line. Consistent with what others and we have reported previously [13,14,15], in this HPV-negative cell line IR effects were potentiated significantly by glycolytic inhibition alone (Figure 4).

## 4. Discussion

In recent years cancer cell metabolism has come to the forefront of cancer research because of increasing links between tumour metabolism and the causal changes determining the cancer phenotype (reviewed in [33]), yet has received relatively little attention in the context of SCCHN generally, and in particular with respect to HPV-positive disease. Renewed interest in this field in cancer research more generally has led to the development of new drugs and re-purposing of existing drugs that target cellular metabolism, which, when combined with standard therapies can offer a selective therapeutic gain (reviewed in [34]). However, the specific nature and extent of the metabolic perturbations can differ significantly between cancers, depending both on genotype and tissue of origin [12]. For instance, although aerobic glycolysis is the best-documented metabolic phenotype, glutaminolysis has been shown to predominate in cervical cancer and glioblastoma [35,36], while enhanced rather than reduced oxidative phosphorylation is a feature of both breast cancer [37] and chronic lymphocytic leukaemia [38], findings that highlight the importance of detailed study of metabolism in cancer site-specific experimental systems.

Until relatively recently metabolic studies in SCCHN were lacking, and for the most part had focused on isolated or limited transporter/enzyme expression, rather than characterising dynamic metabolic flux and presenting a clear picture of the metabolic phenotype (reviewed in [39]). More recent and comprehensive metabolic studies, undertaken in our laboratory and by the Myers’ laboratory, have revealed *TP53* status as a determinant of the metabolic phenotype and therapeutic response in HPV-negative SCCHN [13,15]. These studies, which utilised a similar experimental platform to the present study, demonstrated that cells with compromised p53 function displayed a distinct metabolic phenotype to that retained by wild-type p53 cells. Specifically, wild-type p53 cells displayed robust spare mitochondrial respiratory capacity, while those with compromised p53 function, (regardless of whether this is due to *TP53* mutation or as a consequence of RNAi-mediated down-regulation of wild-type p53), displayed markedly reduced respiratory reserve, functioning near maximal capacity under basal conditions [13,15]. Importantly, this was associated with altered IR response following glycolytic inhibition with 2-DG, which potentiated IR effects in mutant and in cells with RNAi-mediated knock-down of p53 but not in wild-type *TP53* cells [13,15]. Furthermore, these results were reproducible in isogenic cell lines divergent on *TP53* functional status, corroborating a functional dependence on p53 [13,15].

Cognisant of this causal link between loss of p53 function and a less flexible “Warburg” metabolic phenotype, results from the present study are somewhat surprising. It is well documented that HPV, specifically via the viral E6 oncoprotein, inactivates p53 [40], and that as a result *TP53* mutations are highly unusual in HPV-driven tumours of the cervix and also oropharynx (2–3% of cases [16]). Since there is ample evidence that p53 function is compromised in HPV-driven tumours, we might expect that HPV-positive tumour cells would display a similarly inflexible metabolic profile to that observed in SCCHN cells with compromised p53 function. On the contrary, we have found that HPV-positive SCCHN cells display greater metabolic diversity than cells in which p53 function is compromised (either through genetic mutation or experimentally by RNAi), indeed comparable to that observed in p53 wild-type cells [15], and by extension require a combinatorial anti-metabolic approach targeting both mitochondrial respiration and glycolysis to be sensitised to the effects of IR. Although unexpected, these results are broadly consistent with the limited published data on metabolism in HPV-positive SCCHN. In a 2014 study examining metabolic protein expression, increased levels of proteins indicative of oxidative phosphorylation and lower levels of extracellular lactate accumulation were observed in both HPV-positive SCCHN resection specimens and an HPV-positive SCCHN cell line relative to HPV-negative SCCHN models [41]. Similarly, a more recent and robust analysis, including RNA sequencing, gene ontology analysis, and extracellular flux analysis, revealed increased expression of glycolytic genes and higher rates of glycolysis and glycolytic capacity in HPV-negative tumours and cell lines respectively, albeit on a background of a globally altered metabolic profile in HPV-negative disease (including elevated mitochondrial respiration) [42]. Unlike the present study, however, neither of these previous studies examined associated pharmacological manipulation specific to HPV-positive SCCHN.

A highly interesting implication of these data is the suggestion that HPV-positive SCCHN cells display evidence of only partial inhibition of p53. It may be the case that E6-mediated inactivation of p53 is incomplete, leaving sufficient functional wild-type p53 to maintain a balanced and diversified metabolic phenotype. If correct, this raises the intriguing prospect of distinguishing between functions of p53 that are sensitive to E6-mediated inactivation from those that are resistant to this. This could have significant implications for the future management of HPV-positive tumours insofar as treatment strategies may be targeted towards the residual p53 function that we have detected. Consistent with this, genome-wide microarray data from a recent study examining radiation response in HPV-positive and HPV-negative SCCHN cell lines suggested that low levels of wild-type p53 remain in HPV-positive cell lines, that this p53 can be activated by exogenous stress such as IR, and that this effect can be overcome by more complete knockdown of p53 using shRNA [43]. However, whilst our findings of p53 stabilisation and activation of p53 reporters in response to genotoxic stress in one HPV-positive SCCHN cell line (VU-SCC-147) are supportive of this notion, this was not observed convincingly in the other HPV-positive cell lines. Moreover, perhaps the most likely explanation for the observed protein levels relates to the fact that VU-SCC-147 cells harbour a *TP53* mutation leading to a substitution (L257R) [25], which also renders the mutant transcriptionally inactive for, inter alia, MDM2. p53 L257R would be expected to bind to MDM2 but fail to result in upregulation, leading to a potential failure of the auto-regulatory feedback loop. Additionally, specific mutations in the p53 DNA binding domain have been shown to inhibit E6 binding [44,45], and thus the L257R mutation might similarly lead to protein stabilisation, and may indicate another site in this region that alters binding, either indirectly or directly. Whilst this raises the possibility that naturally occurring p53 variants, related to single nucleotide polymorphisms (SNPs), may impact on E6 binding and subsequent p53 degradation, we suggest this is unlikely to be the case. If SNPs, such as the codon 73 Arg/Pro polymorphism, were responsible for differential binding to, and consequently inactivation of, p53, this would be expected to manifest as differential oncogenicity of HPV in individuals with different haplotypes, something for which no reliable association has been detected to date.

We acknowledge several limitations of the work presented here, principally related to the fact that experiments have been conducted exclusively using homotypic, monolayer cell culture of immortalised cell lines derived from SCCHN tumours. Although such cultured cancer cell lines are the most widely used model systems and have formed the basis for much of our current understanding of cancer biology, the clinical relevance of these models has been questioned [46]. One of the major concerns is that of clonal evolution, whereby cells adapt to the culture environment in vitro as they grow and may develop genetic and phenotypic differences from the original tumour [46]. Genetic and molecular cytogenetic data for SCCHN cells lines in culture, however, has shown a close resemblance to those in the primary tumours [22]. In addition, to counteract clonal evolution as best possible, our experiments were performed with cells at relatively early times after thawing from cryopreserved stocks. Nonetheless, to mitigate against this issue more definitively, future pre-clinical experiments could be conducted using short-term, fresh, primary cell cultures. Isolating and propagating such cell lines from HPV-positive SCCHN tumours, however, has proven notoriously difficult. Indeed, whilst there are now thousands of HPV-positive SCCHN and biopsy specimens available, there are only a handful of documented HPV-related SCCHN cell lines available worldwide and there is currently a scarcity of established cell lines with such characteristics, which raises an important question over the validity of the cell lines that do exist, and specifically those used in this study. Recent comprehensive characterisation of these cell lines, however, has revealed integrated and/or episomal viral DNA that is transcriptionally active providing convincing evidence of a persisting viral oncogenic driver [19,20]. However, until E6/7 manipulation of cells is accomplished there will not be a truly reliable link to HPV and functional studies, and this is part of on-going work by other researchers in our laboratory.

A further potential limitation of the work presented here relates to the specificity of the mechanisms of actions of the metabolic inhibitors used. 2-DG is a stable glucose analogue that is taken up by glucose transporters and phosphorylated. 2-DG, however, cannot be fully metabolised and 2-DG-6-phosphate accumulates in the cell and interferes with glycolysis by inhibiting the glycolytic enzymes phosphoglucose isomerase and hexokinase [30,31]. The general assumption, therefore, is that the biological effects of 2-DG are the consequence of this block in carbohydrate catabolism. This assumption, however, has been challenged and any observed effects of 2-DG may not be simply the result of a catabolic block [47]. Nonetheless, the specificity of 2-DG anti-glycolytic effects in suppressing cellular proliferation and anchorage-independent growth in SCCHN cell lines has been demonstrated previously, with effects reproduced under conditions of glucose deprivation [14]. Furthermore, differential sensitivity to halogenated 2-DG analogues consistent with their underlying chemical structure has been reported, and quantitative, broad-based analysis of changes in intracellular metabolite levels in response to 2-DG revealed time-dependent reductions in lactate production and levels of upstream glycolytic and tricarboxylic acid cycle intermediates [14]. Similarly, whilst metformin has well demonstrated activity against electron transport chain complex I [32], multiple other mechanisms of action have been identified including both AMPK-dependent and independent inhibition of mitochondrial respiration, altered fat metabolism, and direct inhibition of fructose-1,6-bisphosphatase [48].

It is widely acknowledged that there is a need for more selective and personalised therapeutic approaches in the wider field of cancer research, and currently much research effort is focused on this key strategy. Such molecularly targeted therapies, however, have as yet had limited translation into the treatment of SCCHN patients and it remains a major challenge to develop such treatments. Novel therapeutic approaches are generally predicated on a discernible therapeutic index of the chosen agent, either in isolation or in combination with other treatment regimes. In contrast to traditional cytotoxic agents, which largely rely on the inherently more incessant proliferative rate of cancer cells rather than true tumour cell specificity, metabolic targeting can exploit the fact that tumour cells become dependent on particular metabolic pathways, providing a selective therapeutic gain while sparing most normal cells. To this end, the findings presented here proffer the opportunity for a tailored anti-metabolic approach to the treatment of HPV-positive SCCHN, whereby these tumours can be sensitised to IR using a combinatorial anti-metabolic approach targeting both mitochondrial respiration and glycolysis. We believe this targeted radiosensitising approach is particularly attractive given the key role of IR as a treatment modality in HPV-positive disease, either as a primary treatment with or without concurrent chemotherapy or as an adjuvant treatment following surgery [49]. Furthermore, this may provide an attractive platform for treatment de-intensification in carefully selected cases by facilitating IR dose reduction to minimise the impact of treatment on long-term function in this ever-increasing patient group.

As alluded to previously, these findings will require further evaluation in the pre-clinical setting prior to translation into clinical practice but do provide rationale for initiating clinical trials. Although no previous clinical studies have been conducted using 2-DG in SCCHN patients, 2-DG in combination with IR has been evaluated more extensively in patients with cerebral gliomas and has been well tolerated with a relatively minor, predictable, and reversible side-effect profile akin to that of hypoglycaemia (reviewed in [50]). Therefore, this data could be extrapolated to inform dosing schedules and safety profiles in SCCHN patients, potentially obviating the need for phase I/II trials before proceeding to phase II/III trials. Similarly, metformin is commonly used in clinical practice as a first line of treatment for type II diabetes with minimal toxicity. Although more specific mitochondrial inhibitors, such as rotenone and cyanide, are available, their associated toxicity makes them unlikely therapeutic options. Indeed, there has been an ever-increasing interest in re-purposing metformin as a therapeutic agent in the context of cancer treatment [51], an interest which stems from several epidemiological studies demonstrating a lower cancer incidence in diabetic patients taking metformin than in the general diabetic population [52], and retrospective data indicating that metformin users have higher rates of disease control when treated with neoadjuvant chemotherapy for breast cancer [53].

## Data Availability

As there are no relevant publicly available data repositories for this work any data is available upon request from the authors.

## Supplementary Materials

The following are available online at https://www.mdpi.com/article/10.3390/cancers13194836/s1, Figure S1: FCCP dose titration for UM-SCC-81B to ensure the reduced mitochondrial reserve was not related to suboptimal FCCP dosing, Figure S2: Original western blots.
