# Supplementary material for: Metabolic Plasticity and Combinatorial Radiosensitisation Strategies in Human Papillomavirus-Positive Squamous Cell Carcinoma of the Head and Neck Cell Lines
Source: Cancers (Basel). 2021 Sep 28;13(19):4836. doi: 10.3390/cancers13194836 (PMC8507998; doi:10.3390/cancers13194836)
Supplement: Supplementary file 1 [file cancers-13-04836-s001.zip › cancers-1390134-supplementary.pdf]

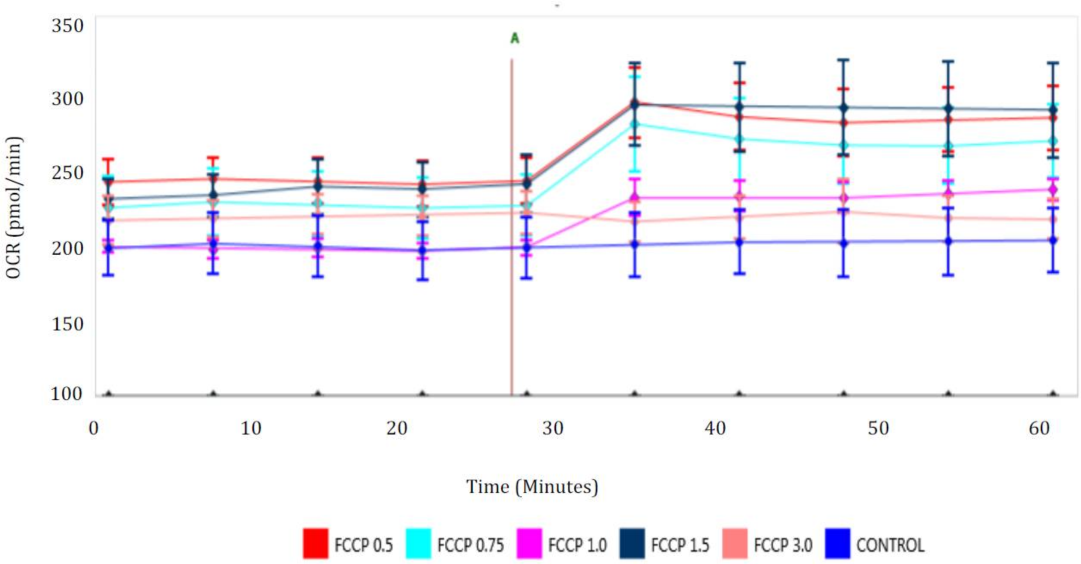

Figure S1. FCCP dose titration for UM-SCC-81B to ensure the reduced mitochondrial reserve was not related to suboptimal FCCP dosing.

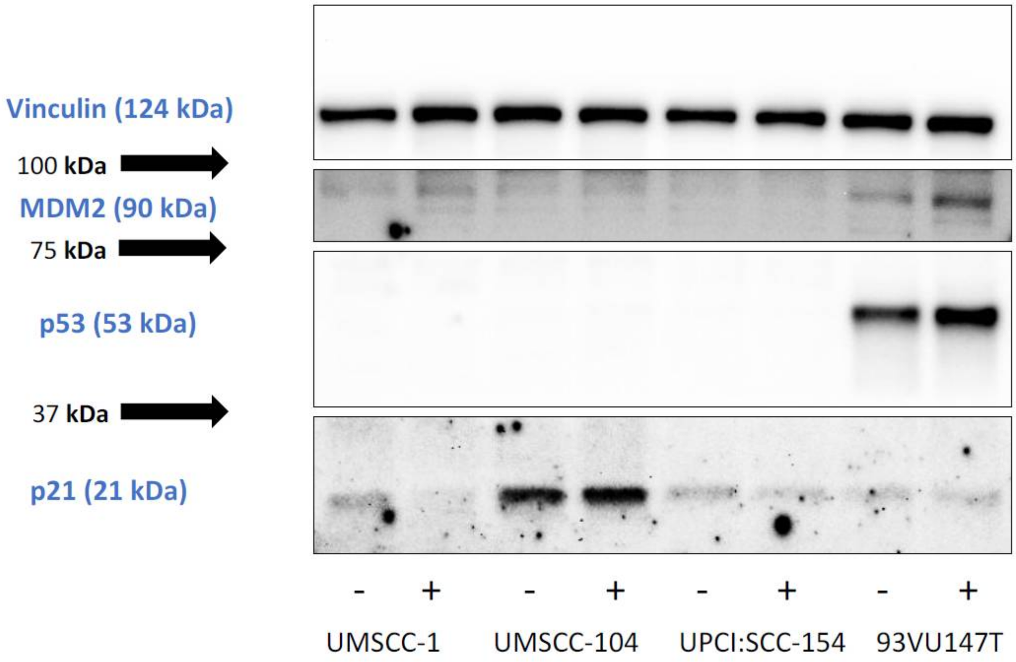

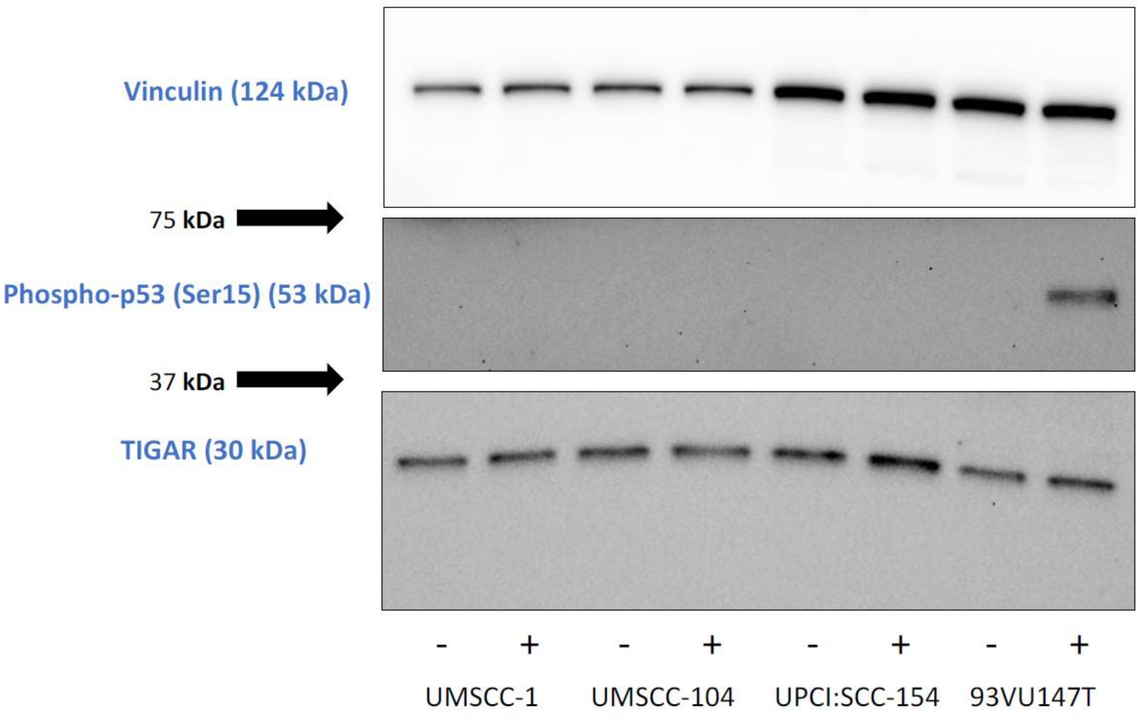

Figure S2. Original western blots.
